# Development of a Three-Dimensional Multi-Modal Perfusion-Thermal Electrode System for Complete Tumor Eradication

**DOI:** 10.3390/cancers14194768

**Published:** 2022-09-29

**Authors:** Hui Zheng, Peicheng Li, Ruidong Ma, Feng Zhang, Hongxiu Ji, Wayne L. Monsky, Evan Johnson, Weizhu Yang, Caifang Ni, Dayong Gao, Xiaoming Yang

**Affiliations:** 1 Image-Guided Biomolecular Intervention Research and Division of Interventional Radiology, Department of Radiology, University of Washington School of Medicine, Seattle, WA 98109, USA; 2 Department of Interventional Radiology, Fujian Medical University Union Hospital, Fuzhou 350001, China; 3 Department of Interventional Radiology, First Affiliated Hospital of Soochow University, Suzhou 215006, China; 4 Department of Mechanical Engineering, University of Washington, Seattle, WA 98195, USA; 5 Department of Pathology, Overlake Medical Center and Incyte Diagnosticsm, Bellevue, WA 98004, USA

**Keywords:** radiofrequency ablation, radiofrequency hyperthermia, mathematic simulation

## Abstract

**Simple Summary:**

Image-guided thermal-ablation techniques, such as radiofrequency ablation (RFA), have become the principal tools for treatment of unresectable primary and secondary tumors. However, tumor recurrences post-thermal ablation of medium to large-sized tumors frequently occur due to incomplete ablation of tumor margins. In the present study, we aim to specifically conquer this clinical problem through revolutionizing current thermal ablation technology by development of an innovative three-dimensional, multi-modal, perfusion-thermal electrode system, with the evidence of potential to simultaneously deliver therapeutics and RFA-medicated peritumoral hyperthermia into the difficult-to-treat tumor margins, to further eradicate the residual tumor cells during the thermal ablation.

**Abstract:**

Background: Residual viable tumor cells after ablation at the tumor periphery serve as the source for tumor recurrence, leading to treatment failure. Purpose: To develop a novel three-dimensional (3D) multi-modal perfusion-thermal electrode system completely eradicating medium-to-large malignancies. Materials and Methods: This study included five steps: (i) design of the new system; (ii) production of the new system; (iii) ex vivo evaluation of its perfusion-thermal functions; (iv) mathematic modeling and computer simulation to confirm the optimal temperature profiles during the thermal ablation process, and; (v) in vivo technical validation using five living rabbits with orthotopic liver tumors. Results: In ex vivo experiments, gross pathology and optical imaging demonstrated the successful spherical distribution/deposition of motexafin gadolinium administered through the new electrode, with a temperature gradient from the electrode core at 80 °C to its periphery at 42 °C. An excellent repeatable correlation of temperature profiles at varying spots, from the center to periphery of the liver tumor, was found between the mathematic simulation and actual animal tumor models (Pearson coefficient ≥0.977). For in vivo validation, indocyanine green (ICG) was directly delivered into the peritumoral zones during simultaneous generation of central tumoral lethal radiofrequency (RF) heat (>60 °C) and peritumoral sublethal RF hyperthermia (<60 °C). Both optical imaging and fluorescent microscopy confirmed successful peritumoral ICG distribution/deposition with increased heat shock protein 70 expression. Conclusion: This new 3D, perfusion-thermal electrode system provided the evidence on the potential to enable simultaneous delivery of therapeutic agents and RF hyperthermia into the difficult-to-treat peritumoral zones, creating a new strategy to address the critical limitation, i.e., the high incidence of residual and recurrent tumor following thermal ablation of unresectable medium-to-large and irregular tumors.

## 1. Introduction

Advances in image-guided interventional technologies have greatly improved the management of malignancies in patients. These efficacious interventional therapies have prominent advantages, including minimal invasiveness, repeatability, low risk of complications, and a short hospital stay. Among various tumor ablative therapies, radiofrequency ablation (RFA) is the most widely investigated and used technique for eradicating small lesions (<3 cm), with comparable overall survival, safety and cost-effectiveness as microwave ablation (MWA) [1,2,3]. However, for medium (3–5 cm) to large (5–7 cm) lesions, both RFA and MWA often create incomplete ablation at the peritumoral zone [4,5]. This incomplete ablation is attributed to several factors, such as the cooling effect of blood flow in the tumor-surrounding vasculatures (namely “heat-sink” effect, e.g., the blood flow of those larger vessels surrounding the radiofrequency (RF)-ablated tumors takes RF thermal heat away, and therefore reduces the treatment efficacy of RF eradication of tumor cells/tissues at the ablated tumor margin), and intentional avoidance of MWA’s rapid over-heating to protect adjacent critical normal structures (such as large vasculatures, bile ducts, diaphragms, or nearby gastrointestinal tracts) [5,6,7,8]. 

**Figure 1:**
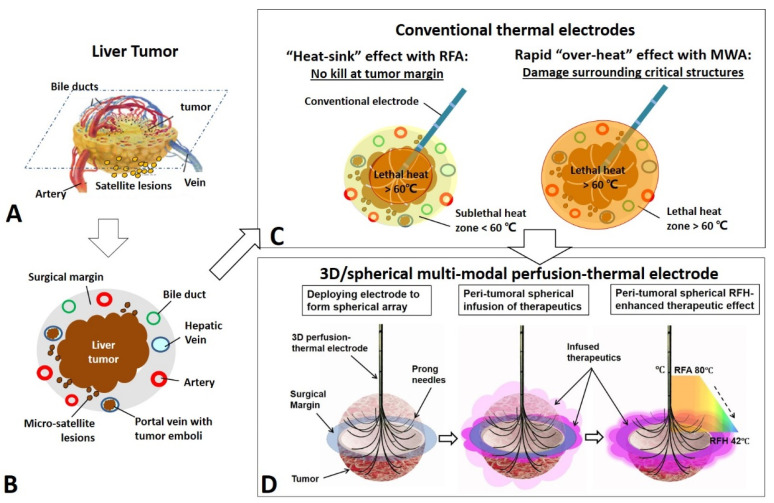
The model for development of the 3D/spherical, multi-modal, perfusion-thermal electrode system for treatment of medium-to-large liver tumors as an example. (**A**) A liver tumor with surrounding structures. (**B**) A cross-sectional view of the liver tumor with infiltrating border, micro-satellite lesions and venous tumor emboli at the 1-cm surgical margin. (**C**) Conventional thermal ablation of medium-to-large tumor suffers from incomplete ablation at tumor periphery, due to either RFA’s “heat-sink” by neighboring blood flow or intentional avoidance of MWA’s “over-heat” to salvage surrounding critical structures. (**D**) The new 3D, multi-modal, perfusion-thermal electrode system provides a “one-stop-shop” solution to overcome such incomplete ablation. The novel spherical perfusion-thermal electrode permits (i) creation of lethal RFA heat (60–100 °C) for necrotic ablation at the tumor center; and (ii) direct spherical infusion of therapeutics into the tumor periphery (surgical margin). The temperature gradient from the central-tumoral RFA lethal heat (e.g., ~80 °C) results in a spherical, peri-tumoral, sublethal hyperthermia (RFH, ~42 °C), which simultaneously increases therapeutic uptake and cytotoxicity, and thereby further enhances destruction of tumor margins.

To address this critical clinical problem, we proposed to simultaneously deliver anti-tumor agents to the tumor periphery during an ablative therapy session. This concept combines the current conventional thermal ablation, with lethal heat (>60 °C) to destroy tumor cells in the tumor center, with specific peritumoral delivery of high-dose therapeutics to further kill tumor cells within the peritumoral zone resulting in complete eradication of all viable tumor cells in and adjacent to lesions, while sparing surrounding normal structures from overheating by current thermal ablation technique (Figure 1). 

Recent studies from our group and others have confirmed that image-guided radiofrequency hyperthermia (RFH, at a sub-lethal temperature <60 °C) can enhance gene-, chemo-, and immunotherapy of malignancies in various organs [9,10,11,12]. The recognized mechanisms of RFH-enhanced therapies include heating-induced tissue fracture, increased permeability of cytoplasmic membranes, disruption of cellular metabolism, activation of membrane-associated pumps, and activation of heat shock protein pathway [13,14,15,16,17,18]. These mechanisms effectively facilitate the entry of therapeutic agents into tumor cells once they arrive at the extracellular space of the target tumors by either local or systemic delivery, and thereby promote the destruction of tumor tissues.

These groundworks lead to our current innovation, developing a novel three-dimensional (3D) spherical, multi-modal perfusion-thermal electrode system, to effectively treat peripheral tumor cells by creating a clean surgical margin, which thus specifically addresses the critical clinical limitation of thermal ablation therapies, i.e., the high incidence of residual and recurrent tumor following thermal ablation of unresectable medium-to-large tumors.

## 2. Materials and Methods

### 2.1. Study Design 

This study was implemented in five steps: (1) design of the 3D/spherical multi-modal perfusion-thermal electrode system; (2) production of this system; (3) ex vivo evaluation of its capability/functionality; (4) confirmation of its accurate temperature profiles using mathematic modeling/computer simulation; and (5) in vivo validation of its technical feasibility using living animal models with orthotopic hepatic tumors.

### 2.2. Design/Production of the 3D/Spherical, Multi-Modal, Perfusion-Thermal Electrode System 

**Figure 2:**
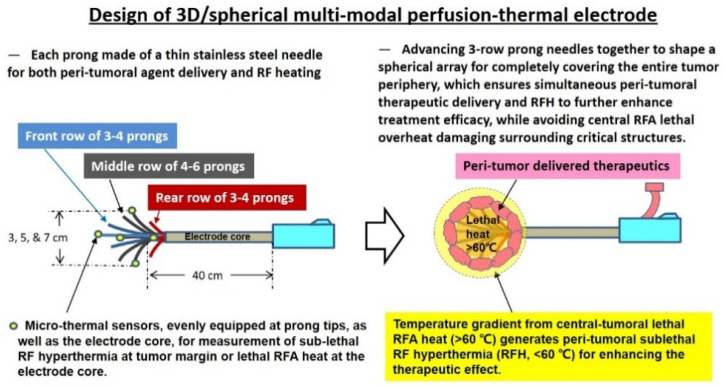
Design of the 3D/spherical, multi-modal, perfusion-thermal electrode system, which is characterized by its spherical array made up of 3-rows of thin and hollow prong needles for simultaneous peritumoral delivery of high-dose therapeutics and RF hyperthermia (RFH), while maintaining RFA lethal necrotic heat at the tumor center.

We specifically designed the new multi-modal, perfusion-thermal RFA electrode, with (1) multiple hollow prong needles to be deployed from the electrode core, to form a 3D spherical array at adjustable sizes of 1–3, 3–5, or 5–7 cm^3^; (2) the lengths of prong needles at 1.5, 2.5 or 3.5 cm, and; (3) each hollow prong needle having a 0.3-mm inner diameter channel for simultaneous delivery of therapeutics into the ablated tumor periphery. Thermal couple micro-thermosensors were integrated into some prongs, which enabled the real-time monitoring of temperature change and thereby, precisely controlled the spherical hyperthermia distribution from the expected prong arrays (Figure 2). 

### 2.3. Ex Vivo Evaluation

#### 2.3.1. Evaluating the Function of Peripheral Agent Delivery

The whole fresh pig liver was placed in a 37 °C water bath. Under ultrasound (US) imaging guidance, the 3D, perfusion-thermal electrode was positioned in the liver, with the electrode connected to an RF generator for RF heating. After fully opening the multiple prongs under US guidance, a 2-mL mixture (1:1) of trypan blue (Life Technologies, Carlsbad, CA, USA) and motexafin gadolinium (MGd, Pharmacyclics, Sunnyvale, CA, USA) was infused via the electrode prong needles. The trypan blue stain was used for pathologic confirmation, while MGd with its distinguishing property of emitting red fluorescence used for optical imaging (In-vivo Xtreme; Bruker, Billerica, MA, USA) at 470-nm excitation, 750-nm emission, 90 × 190 mm^2^ field, and 30-s exposure time.

#### 2.3.2. Evaluating the Function of Stable RFA/RFH Heat Generation

Another whole fresh pig liver was placed in a 37 °C-water bath. After placement of the new electrode into the liver, fiberoptic micro-thermosensors were positioned, in parallel, to simultaneously measure the temperature gradient at different distances of 0, 0.5, 1, 1.5, 2, and 3 cm from the electrode core. The working frequency of RF device was 500 kHz. The central RFA-induced lethal temperature rise was precisely monitored/recorded on the digital monitor panel of the RFA system, while the peripheral sublethal temperature around the deployed prong array of the electrode was measured by the micro-thermosensors connected to its thermometer (PhotonControl, Burnaby, BC, Canada). 

### 2.4. In Vivo Validation

#### 2.4.1. Creation of Animal Models with Orthotopic Hepatic Tumors

Animal experiments were approved by the institutional animal care committee of University of Washington. Five adult, female, New Zealand White rabbits (weighed at 2.5–3 kg) with orthotopic VX2 liver tumors were created using our established protocol [19]. 

#### 2.4.2. The Mathematic Modeling and Computer Simulation of the 3D Multi-Modal Electrode Performance

**Figure 3:**
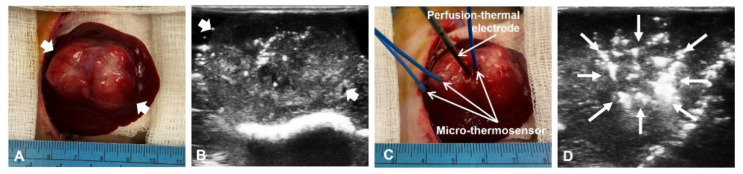
In vivo experimental set up. (**A**) A hepatic VX2 tumor (between arrows) in a living rabbit is successfully created and detected with ultrasound imaging (**B**). (**C**) The 3D multi-modal electrode is inserted into the tumor, while three micro-thermosensors positioned at different points across the tumor, including the center (the electrode core), the middle (between the center and tumor margin), and at the tumor margin. (**D**) The intratumoral positioning of the electrode, as well as deploying the appropriate array of its prong needles (arrows) are precisely monitored and controlled under real-time ultrasound imaging guidance.

To evaluate and predict the thermal energy-producing feature of the new 3D electrode system, we employed a mathematic simulation model with the tumor sizes specifically larger than 3 cm, which thus enabled us to understand the actual thermal mapping of each prong and energy-distribution patterns of the new electrode. The temperatures obtained by mathematic simulation were verified by the actual temperatures measured in rabbit models with orthotopic liver VX2 tumors larger than 3 cm. Three fiberoptical micro-thermosensors (Micronor LLC, Camarillo, CA, USA) were placed at different points, i.e., the electrode core (center), middle (between center and tumor margin), and at the tumor margin for real-time temperature measurements (Figure 3). The real temperature changes were recorded for 20 min as the central RFA heating (>60 °C) was applied to the tumor, while the standby power of the whole RFA device was about 44–45 watts.

We used a COMSOL (COMSOL Inc., Burlington, MA, USA) Multiphysics software to establish a finite element method model (Figure S1). RFA relied on Joule effect heating (fractional heating). After the placement of the electrode and grounding pad on the target, RF generator is applied to the electrode with a certain power, and the ion flow tends to follow in the direction of AC current [20,21]. With a high frequency of AC current, the target tissues, not the electrode, produce frictional heating and induce coagulation and cellular necrosis in the tumor when the temperature reaches a certain level, typically above 60 °C [22,23]. Firstly, the bio-heat transfer equation for the determination of temperature distribution profile in the tumor and the 3D perfusion-thermal electrode system is described by the Pennes equation as follows [23]:

(1) $$\rho C_{p}\frac{\partial T}{\partial t}+\nabla \cdot q=Q_{bio}+Q_{e}$$

where ρ is the tumor tissue density (*kg/m^3^*); $C_{p}$ is the tumor tissue specific heat capacity (*J/kg∙K*); *T* is the temperature; *q* is the heat flow; *Q_bio_* is the overall heat source including fluid/blood flow perfusion loss and metallic heat generation. The heating process of RFA was simplified to a conduction heat transfer since the heat generated inside tumor tissue dissipated along the normal tissue. *Q_e_* (*A/m^3^*) is a heat source term due to the resistive heating (Joule heating), which includes the resistive losses and magnetic losses; the magnetic losses are too small and are ignored.

Secondly, the RF equation was governed by the applied voltage or current. The electric field was distributed into the biological tissue from the electrode prongs and the current field is regarded as heating source governed by a generalized Laplace equation:

(2) $$\nabla \cdot \left(\sigma \nabla V\right)=0\;$$

where σ is the electrical conductivity of the material (S/m), a measure of how easily charges move through it; *V* is voltage source (*V*), which related to resistance and power. Regarding the quasi-static approximation of Maxwell’s equation, the RFA electrical field and current density are defined as:

(3) $$\vec{E}=-\nabla V$$

(4) $$\vec{J}=\sigma \;\vec{E}$$

$\vec{E}\;$ is the electric field vector *(V/m)*; $\vec{J}$ is the current density vector. The overall heat generation of *Q_e_* is computed as:

(5) $$Q_{e}=\vec{J}\cdot \vec{E}=\frac{\;1}{2}\sigma {\left|\vec{E}\right|}^{2}$$

The mathematic simulation model was studied under quasi-static approximation. The values of important material properties used in the computer simulation are shown in Table 1.

#### 2.4.3. Treatment of VX2 Hepatic Tumors

Approximately 3 weeks after VX2 hepatic tumors implantation, we placed the 3D perfusion-thermal electrode, under real-time US guidance, into the tumor, and then deployed the prong needles at its appropriate array to create a full spheric coverage of the target tumor. Through the prong hollows, we delivered 3–5 mL an FDA-approved optical imaging dye, indocyanine green (ICG, 100 μg/ mL, Patheon Italia S.P.A, Ferentino, Italy) into the tumor periphery, while using the same device to generate the lethal temperature (>60 °C) in the tumor center and the sub-lethal temperature (<60 °C) at the peripheral zone for 20 min to enhance the uptake of ICG by VX2 tumor cells. For evaluating the temperature correlation between the mathematic simulation model and the actual living animal model, three fiberoptic micro-thermosensors were placed to monitor the temperatures at different locations of the tumor center (the electrode core), the middle point between the tumor center and the tumor periphery, and the tumor periphery (defined as the boundary between tumor margin and surrounding normal liver parenchyma).

### 2.5. Optical Imaging of Liver VX2 Tumors

We euthanized animals 24 h after the treatments, harvested the entire tumor-containing livers for optical imaging, and then sectioned the livers at 5-mm thickness for optical “tomography”, at 760-nm excitation, 830-nm emission, 120 × 120-mm^2^ field of view, and 1-min exposure time (In-vivo Xtreme; Bruker, Billerica, MA, USA) [24]. 

#### Pathology Correlation/Confirmation 

The sectioned tumor-containing liver was flash-frozen and further serially cryosliced at 10-μm thickness. Different histologic staining of the serial slides were performed, including (i) counterstaining with 4’,6-diamidino-2-phenylindole (DAPI; SouthernBiotech, Birmingham, AL, USA) for fluorescent microscopy (IX73, Olympus, Tokyo, Japan); (ii) nicotinamide adenine dinucleotide hydrogen (NADH) staining for histological examination of the ablation necrosis; (iii) hematoxylin and eosin (H&E) staining for confirmation of the tumor formation and peritumoral infiltration; and (iv) immunohistochemical staining for heat shock protein 70 (HSP70) to determine the potential activation mechanism for RF hyperthermia.

### 2.6. Statistical Analysis

Statistical analyses were performed using Origin (Origin Lab Corporation, Version 2022b) and SPSS Statistics (IBM Corp. Version 27.0.), while the Pearson coefficient/ICC analysis used to determine the correlation between the measured temperatures of the actual living animal tumor model and the corresponding temperatures were calculated/predicted by mathematic simulation model.

## 3. Results

### 3.1. Ex Vivo Evaluation

**Figure 4:**
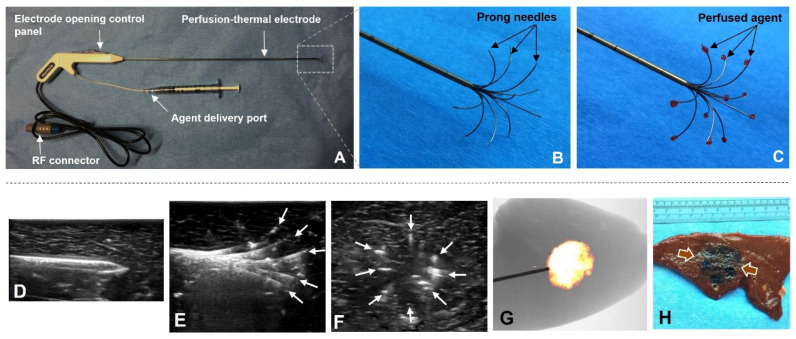
Production of the new 3D/spherical, perfusion-thermal electrode system and confirmation on successful peripheral delivery of the agent. (**A**) The perfusion-thermal electrode system. (**B**,**C**) The prototype of the new 3D, perfusion-thermal electrode. The spherical array of prong needles is adjustable at a range of various sizes, and, thereby, can completely cover the tumor margin zone for peri-tumoral spherical delivery of therapeutic agents (pink). (**D–F**) Positioning of the electrode and deploying of the prong needles (arrows on E, at longitudinal view, and; F, at cross-sectional view) can be precisely monitored under real-time ultrasound imaging guidance. (**G**) Optical imaging detects a MGd-emitted light ball, which is further confirmed by the cross pathology as a spherical blue stain (between arrows) (**H**).

We specifically designed the new multi-modal, perfusion-thermal RFA electrode system, with its prong needles configured in a spherical 3D array, which thus enabled the entire therapeutic coverage of the ablated tumor by not only peritumoral delivery of high-dose therapeutics, but also peritumoral generation of sublethal RFH (<60 °C) along with the heating gradient from the central tumoral RFA lethal heat (>60 °C) (Figure 4). In evaluating the peripheral perfusion function, the expected array of the electrode prongs was precisely monitored by real-time US, while optical imaging demonstrated the distribution of the peripherally-delivered MGd as a spherical shell, which was further evidenced by gross pathology as outlined with trypan blue stain (Figure 4).

**Figure 5:**
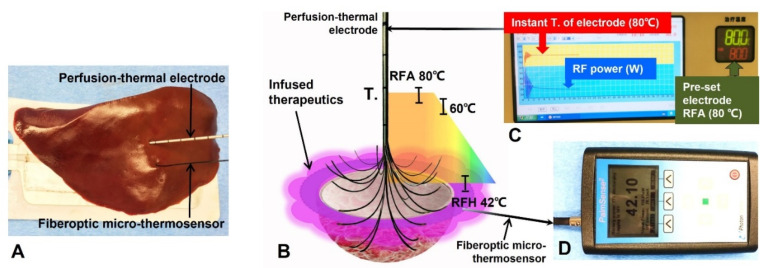
Confirmation of generating a stable peripheral sublethal RFH from gradient of the central lethal RFA heat. (**A**) Experimental set-up. After placement of the multi-modal electrode, a fiberoptic micro-thermosensor positioned, in parallel, to simultaneously measure the temperature gradient at different distances from the electrode core. (**B**) Results of measured temperature (T) gradient of the perfusion-thermal electrode. (**C**) The digital monitor panel of the new RFA system, demonstrating both the central RFA lethal heat (red) and RF power (blue) are consistent throughout the 10-min heating, which is precisely monitored on the panel. When pre-setting the central RFA lethal heat at 80 °C (green on C), a peripheral sublethal RFH at ~42 °C is recorded by the fiberoptical micro-thermosensor (**D**).

For the function of peripheral hyperthermia as created when setting the lethal temperature of the electrode core at 80 °C (80-watt RF power for 20 min), we achieved a stable and linear temperature gradient across the temperature measurement points with a peripheral sublethal RF hyperthermia at approximately 42 °C within a 1.5 cm distance (larger than the 1-cm surgical margin of a thermal ablated tumor) from the electrode prong tips (Figure 5). 

### 3.2. In Vivo Validation 

**Figure 6:**
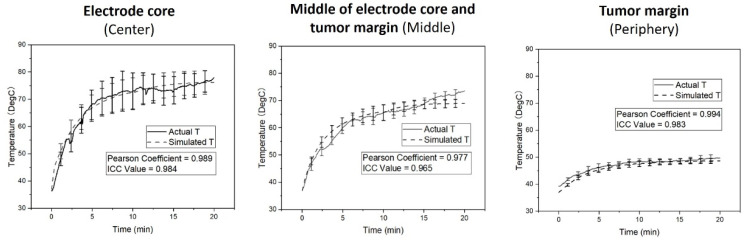
Temperature distribution (mean ± SD) between the actual measurement and mathematic simulation at the three positions under five groups. An excellent correlation is shown between the mathematically simulated temperatures and actual temperatures measured in liver tumors at the center, the middle between the center and the margin, and at the tumor margin, respectively.

All VX2 hepatic tumors at an average size of 3.31 ± 0.35 cm were successfully created in rabbit livers (Figure 3). Overall treatment power of RFA device was 85.53 ± 5.69 watts at its frequency of 500 kHz. The simulation model demonstrated that the temperatures at the tumor center, tumor middle location, and the tumor margin reached the steady states of 77.82 ± 3.81 °C, 73.54 ± 1.78 °C and 49.64 ± 1.37 °C (mean ± SD), respectively (Figure 6). The plot of the actual temperatures measured at different time points of the hepatic tumors showed the same elevation pattern as the temperature changes calculated by mathematic simulation, with the coefficients by the Pearson correlation analysis were close to 1 at all three temperature measurement positions (Figure 6). Intraclass correlation (ICC) analysis at various positions were 0.984 at the center (95% confidence interval, 0.982–0.985), 0.965 at the middle (95% confidence interval, 0.961–0.969), and 0.983 at the tumor margin (95% confidence interval, 0.981–0.985) (Figure 6).

**Figure 7:**
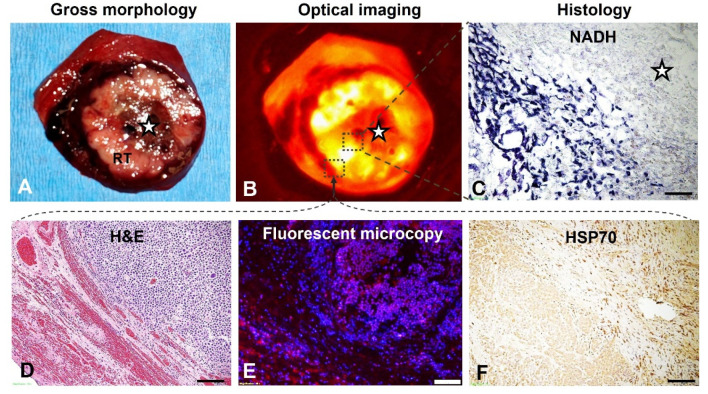
Gross pathology and optical imaging, with histology confirmation using different staining. (**A**) The sectioned-gross specimen of the VX2 tumor-containing liver demonstrates the central tumoral necrosis (stars) due to lethal RFA ablation with the intently-created peripheral residual tumor (RT). (**B**) Optical imaging detects an ICG-emitted florescent signal ring (yellow) at the tumor periphery, indicating the successful peritumoral delivery/deposition of ICGs, in contrast to the ablated central tumor zone with no ICG signals. (**C**) NADH staining confirms the un-stained necrosis at the central tumoral ablated zone, with peripheral residual tumor (blue). (**D**) H&E staining of the tumoral periphery shows the residual tumor cells (blue dots) infiltrating to the surrounding liver parenchyma. (**E**) Fluorescence microscopy further confirms the successful distribution/deposition of ICGs (pink) at the peripheral residual tumor and the margin of surrounding liver parenchyma, which establishes the “proof-of-principle” for further applying therapeutics to eliminate residual tumor cells at the incompletely ablated tumor periphery by using the new 3D, multi-modal electrode system. (**F**) HSP70 staining demonstrates gradient distribution of brown-stained cells in the tumor periphery, indicating the successful creation of peritumoral hyperthermia via the HSP70 pathway. (Scale bars, 20 μm).

Gross pathology of sectioned tumor specimen demonstrated the central coagulative necrosis and residual viable tumor tissue at the peripheral zone. The optical imaging of the same section detected an ICG-emitted florescent signal ring from the tumor periphery, indicating successful peritumoral delivery/deposition of ICG. (Figure 7). The manifestation of optical imaging was further confirmed with fluorescence microscopy, demonstrating the successful distribution/deposition of ICGs at peripheral residual tumor and surrounding liver parenchyma. In addition, pathology examinations with different staining further demonstrated the un-stained ablative necrosis at the tumor center by NADH staining, and the incompletely ablated tumor periphery by H&E staining. Immunohistochemical staining for HSP70 expression displayed the gradient distribution of brown-stained cells at tumor periphery, further indicating and confirming the successful creation of peritumoral RF hyperthermia, which was activated through the traditional HSP70 pathway (Figure 7). 

## 4. Discussion 

A clear “surgical or safety margin” of the ablated lesion periphery is the pivotal factor that warrants a successful tumor ablation. In this study, we specifically designed the new 3D multi-model perfusion-thermal electrode system, which has the following unique advantages:

1. The 3D/spherical multi-modal perfusion-thermal electrode provides the evidence on the potential to direct infuse therapeutics at the ablated tumor periphery, to ultimately destroy micro-satellite lesions, micro-venous tumor emboli and occult invasion that often occur at the difficult-to-treat peritumoral zone when using current thermal ablation devices [25,26].

2. The new multi-modal electrode has the unique characteristic of creating a temperature gradient from the conventional central lethal RFA heat (>60 °C) to the peritumoral sub-lethal RFH (<60 °C), which has been shown to provide further evidence of the potential to improve the intracellular uptake of cytotoxic therapeutics, thereby further enhancing the killing of residual tumor cells in the ablated tumor periphery.

3. Under real-time imaging guidance, a single precise position of the 3D perfusion-thermal electrode in the tumor avoids multiple rounds of electrode placements, currently required when treating a medium-to-large sized lesion with current conventional thermal ablation, thus reducing the overall procedure time [27]. 

4. The single-session treatment using the 3D/spherical perfusion-thermal electrode system to simultaneously deliver therapeutics and hyperthermia can replace the current strategy of combining RFA with additional trans-arterial chemoembolization or radioembolization, thereby reducing the risk of complications and iatrogenic tumor spread due to multiple manipulations [28,29,30].

5. Ultimately, this “one-stop-shop” innovation should fulfill our goal of completely eradicating medium-to-large tumors, while sparing surrounding critical structures from high lethal heat by current thermal-ablation techniques. 

In this study, via serial ex vivo and in vivo experiments we have validated and successfully confirmed the simultaneous functions of the new electrode system, i.e., infusion of therapeutics at the tumor periphery/margin and peripheral sublethal hyperthermia. For evaluating efficacy, we performed in vivo validation with orthotopic hepatic tumors. Both optical imaging and fluorescent microscopy confirmed the successful peritumoral distribution/deposition of the delivered agents, from the ablated tumor periphery to its surrounding liver parenchyma via the 3D spherical prong needles. Thus, these results have established the “proof-of-principle” for further applying different therapeutics (chemo-, gene-, and immuno-therapeutics) to eliminate residual tumor cells at the difficult-to-treat periphery of the ablated medium-to-large lesions. Future studies will aim to optimize the perfusion parameters (such as total infusion volumes and flow rates) for peritumoral delivery of sufficient therapeutics.

The appropriate design of the 3D multi-model electrode system was further confirmed by the mathematic simulation, with evidence of the close temperature correlations between the mathematic simulation model and the actual living animal model, which were consistent at different points along the central tumoral RFA-mediated heat gradient to the peritumoral RFH. These results will encourage us to further establish a pre-RFA planning formula through mathematical modeling and computer simulation for predicting the relationship among the tumor size, electric feeding power, and optimized ablation parameters associated with optimized peritumoral delivery of high-dose therapeutics, which thus provides clinicians a time- and cost-saving approach for even more effective treatment of those patients with unresectable medium-to-large malignancies using the 3D, multi-modal, perfusion-thermal ablation technology.

## 5. Conclusions

We present a new 3D/spherical, multi-modal, perfusion-thermal electrode system, which provides the evidence on the potential to enable direct delivery of agents with simultaneous generation of RFA-associated RF hyperthermia in the peritumoral zones. This innovative design and application of the new electrode system may provide an effective and promising solution to address the inherent pitfalls of current conventional thermal ablation techniques that are often incapable of inducing complete tumor eradication of unresectable medium-to-large malignancies.

## Figures and Tables

**Table 1:** Thermal and electrical Properties (500 kHz).

Material	σ (*S/m*)	*C_p_* (*J/kg∙K*)	*k* (*W/m∙K*)	*ω_b_* (*s^−1^*)
Liver	0.333	3600	0.512	0.0017
Tumor	0.1168	4200	0.552	0.0156
Prongs	9.8 × 10^5^	500	36.7	
Trocar	10^−16^	1010	0.23	
Blood		4180		0.0064

Where σ is the conductivity of the material *(S/m)*, a measure of how easily charges move through it; $C_{p}$ is the specific heat capacity (*J/kg∙K*); *k* is the thermal conductivity *(W/m∙K);* $\omega_{b}$ is the fluid/blood perfusion rate (*1/s*).

## Data Availability

The data presented in this study is available in this article (and supplementary material).

## Supplementary Materials

The following supporting information can be downloaded at: https://www.mdpi.com/article/10.3390/cancers14194768/s1, Figure S1: The configuration of the electrode prongs and tumor.
