# Supplementary material for: Development of a Three-Dimensional Multi-Modal Perfusion-Thermal Electrode System for Complete Tumor Eradication
Source: Cancers (Basel). 2022 Sep 29;14(19):4768. doi: 10.3390/cancers14194768 (PMC9562205; doi:10.3390/cancers14194768)
Supplement: Supplementary file 1 [file cancers-14-04768-s001.zip › cancers-1890417-supplementary.pdf]

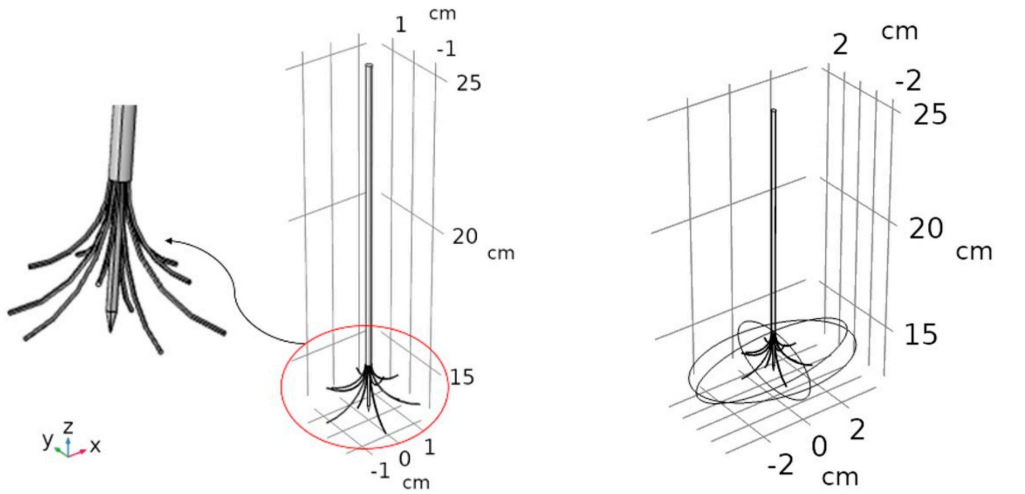

Figure S1. The configuration of the electrode prongs and tumor. The 3D multi-model RF electrode is composed of 11 prongs containing 5 long-curved prongs, 5 short-curved prongs, and 1 straight electro core.
